# Gait-Related Brain Activity in People with Parkinson Disease with Freezing of Gait

**DOI:** 10.1371/journal.pone.0090634

**Published:** 2014-03-03

**Authors:** Daniel S. Peterson, Kristen A. Pickett, Ryan Duncan, Joel Perlmutter, Gammon M. Earhart

**Affiliations:** 1 Program in Physical Therapy, Washington University in St. Louis, St. Louis, Missouri, United States of America; 2 Department of Neurology, Washington University in St. Louis, St. Louis, Missouri, United States of America; 3 Anatomy and Neurobiology, Washington University in St. Louis, St. Louis, Missouri, United States of America; 4 Radiology, Washington University in St. Louis, St. Louis, Missouri, United States of America; Duke-NUS Graduate Medical School, Singapore

## Abstract

Approximately 50% of people with Parkinson disease experience freezing of gait, described as a transient inability to produce effective stepping. Complex gait tasks such as turning typically elicit freezing more commonly than simple gait tasks, such as forward walking. Despite the frequency of this debilitating and dangerous symptom, the brain mechanisms underlying freezing remain unclear. Gait imagery during functional magnetic resonance imaging permits investigation of brain activity associated with locomotion. We used this approach to better understand neural function during gait-like tasks in people with Parkinson disease who experience freezing- “FoG+” and people who do not experience freezing- ”FoG−“. Nine FoG+ and nine FoG− imagined complex gait tasks (turning, backward walking), simple gait tasks (forward walking), and quiet standing during measurements of blood oxygen level dependent (BOLD) signal. Changes in BOLD signal (i.e. beta weights) during imagined walking and imagined standing were analyzed across FoG+ and FoG− groups in locomotor brain regions including supplementary motor area, globus pallidus, putamen, mesencephalic locomotor region, and cerebellar locomotor region. Beta weights in locomotor regions did not differ for complex tasks compared to simple tasks in either group. Across imagined gait tasks, FoG+ demonstrated significantly lower beta weights in the right globus pallidus with respect to FoG−. FoG+ also showed trends toward lower beta weights in other right-hemisphere locomotor regions (supplementary motor area, mesencephalic locomotor region). Finally, during imagined stand, FoG+ exhibited lower beta weights in the cerebellar locomotor region with respect to FoG−. These data support previous results suggesting FoG+ exhibit dysfunction in a number of cortical and subcortical regions, possibly with asymmetric dysfunction towards the right hemisphere.

## Introduction

Gait dysfunction is common in Parkinson disease (PD), and includes short steps [1], increased step time variability [2], and poor step-to-step coordination [3]. Furthermore, about 50% of people with advanced PD also experience Freezing of Gait (FoG) [4], [5], defined as a transient inability to complete effective stepping [6]. FoG is a disabling and distressing symptom, contributing to falls and reduced quality of life [5], [7]–[10], and common PD treatments such as anti-Parkinson medication do not consistently provide adequate benefit [11]. Although FoG is transient, people who experience freezing (FoG+) may exhibit altered gait even during normal walking (i.e. periods of non-freezing or festination), suggesting that the underlying pathophysiology also affects non-freezing locomotion [3], [12], [13].

The neural underpinnings of freezing of gait remain unknown. Two recent reports used functional magnetic resonance imaging (fMRI) to investigate brain activity in FoG+ during gait-like tasks [14], [15]. These reports showed FoG+ to exhibit altered activity in the mesencephalic locomotor region (MLR) compared to people who do not freeze (FoG−) during gait imagery [14], and during lower limb motor blocks [15]. Together, these results support the notion that altered activity in brainstem regions may relate to freezing of gait. In addition, Shine and colleagues also showed reduced activity in the globus pallidus, putamen, and several cortical areas during motor arrests in FoG+. These studies, along with many others [14], [16]–[24], have shown the efficacy of using gait-like tasks, including gait imagery, to assess locomotor dysfunction. This technique relies on the substantial overlap in brain activation responses during imagined and overt movements [25]–[28] including walking [28], [29]. Despite limitations, this approach has provided important insight into brain activation during locomotion in humans [16], [17], [30].

Previous gait imagery tasks used to compare FoG+ and FoG− focused on imagined forward walking. However, more complex gait tasks such as turning increase freezing risk and gait dysfunction [11], [13], [31], [32]. Therefore, brain dysfunction in those who freeze may be more pronounced during these tasks than during forward walking. The underlying mechanisms of increased dysfunction during complex gait are not well understood, yet asymmetry and reduced coordination of steps during complex gait tasks, such as turning, may precipitate freezing [33]. Turning necessitates asymmetries in step length and leg velocity [34], and leads to discoordinated stepping in people with PD. Further, turning by walking in large rather than small circles provides a clinical strategy to improve coordination and reduce freezing [13], [35]. Together, these data suggest the possibility that increased freezing during complex gait tasks such as turning may be due to the inherent asymmetry and discoordination present during these movements.

Therefore, the purpose of this study is to investigate differences in brain activity in FoG+ and FoG− during simple and complex gait tasks. We measured BOLD response during imagery of simple (forward) and complex (backward walking, turning) gait, as well as imagined standing in FoG+ and FoG−. We hypothesized that FoG+ would have altered BOLD responses with respect to FoG− during imagined gait tasks in the following locomotor regions of interest (ROIs): supplementary motor area (SMA), globus pallidus (GP), putamen, MLR, and cerebellar locomotor region (CLR). Further, we expected imagery of complex tasks (turning, backward walking) compared to forward walking would enhance these differences.

## Methods

### 1 Ethics Statement

This protocol was approved by the Washington University in St. Louis internal review board. Written informed consent was provided by all subjects in accordance with the Human Research Protection Office and the Declaration of Helsinki.

### 2 Participants

Inclusion criteria included diagnosis of idiopathic PD as described by Racette et al. [36] and based on established criteria [37], no lower limb injuries for the previous 6 months, no contraindications for MRI, and ability to effectively imagine movement based on the Kinesthetic Visual Imagery Questionnaire (KVIQ) [38]. Thirty three individuals were screened, and those with an average score less than 3 on either the kinesthetic or visual component of the KVIQ, indicating moderate clarity and intensity of imagery, were excluded. Seven individuals with PD were excluded (no fMRI data were collected) based on this imagery vividness threshold. All participants also completed the Gait Imagery Questionnaire [39] (GIQ) to permit post-hoc comparisons of ability to imagine gait across groups, though this score was not used to exclude participants (Table 1). Exclusion criteria included neurological problems other than PD and cognitive dysfunction (Mini Mental State Exam; MMSE<27).

**Table 1:** Participant characteristics.

	FoG−	FoG+	p-value
**N**	9 (7 male)	9 (5 male)	–
**Age (yrs)**	62.7 (8.5)	66.6 (6.7)	0.29
**MDS-UPDRS-III** #	27.7 (8.8)	36.1 (9.3)	0.07
**Hoehn & Yahr**	2.22 (0.26)	2.5 (0.35)	0.08
**Years since Diagnosis**	3.6 (3.1)	9.4 (7.2)	**0.04**
**Preferred Walking Speed (m/s)**	1.0 (0.1)	0.90 (0.2)	0.22
**MMSE** +	28.2 (1.2)	28.6 (1.1)	0.55
**NFOG-Q total score** $	–	13.0 (8.2)	–
**KVIQ** *	81.4 (11.8)	74.3 (12.4)	0.22
**GIQ** *	28.1 (4.4)	25.3 (10.8)	0.33

# Movement Disorders Society Unified Parkinson's Disease Rating Scale (part III).

+ Mini Mental State Exam.

$ New Freezing of Gait Questionnaire.

*KVIQ: Kinesthetic Visual Imagery Questionnaire, max score 100.

*GIQ: Gait Imagery Questionnaire, max score 40.

One left handed participant was included in the FoG+ group.

Individuals were classified as those who experience freezing “FoG+”, and those who do not experience freezing “FoG−” using the New Freezing of Gait Questionnaire (NFOGQ) [40]. People who identified themselves as FoG+ in question 1 went on to answer 8 questions assessing the severity of freezing and its effects on daily life. All data collection was conducted after a 12-hour withdrawal of anti-Parkinson medication. FoG+ and FoG− were matched as closely as possible for disease severity level. Motor severity was assessed by the motor subscale of the Movement Disorders Society Unified Parkinson's Disease Rating Scale (MDS-UPDRS part III).

### 3 Procedure

Participants were first trained to complete five overground tasks: forward walking, backward walking, turning to the left and right in small radius (r = 0.6 m) circles, and standing quietly. Participants were instructed to walk at a natural, comfortable speed for each task. Participant completed each task at two different distances (4 and 8 m for forward and backward gait; 2 and 3 revolutions for turning). The time necessary to complete each gait task was recorded. Training lasted approximately 20 minutes, in which participants completed each task a minimum of 2 times. Participants also practiced imagining each task.

Participants then completed two T2*-weighted gradient echo multislice sequence scans (EPI, TR = 2200 ms, TE = 3 ms, 4.0 mm^3^ voxels, FA = 90°, 9:45 min). BOLD signal was captured for 36 slices covering the brain and the cerebellum. A T1-weighted sagittal, magnetization prepared rapid acquisition with gradient echo (MP-RAGE, TR = 2400 ms, TI = 1000 ms, TE = 3.16 ms, FA = 8°, 1.0 mm^3^, 8:09 min) scan was also collected for identification of ROIs and co-registration of the T2* scans. MR was done with a Siemens 3T Magnetom TrioTim scanner. After the fMRI scans, participants underwent an informal exit interview in which they were asked if they experienced any freezing episodes during any imagery bouts.

**Figure 1:**
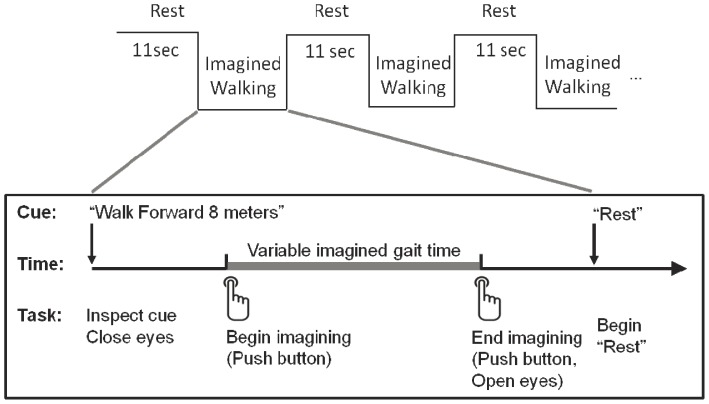
Gait imagery task. After reading the cue, the participant closes his eyes, pushes a button, and begins imagining. At the completion of gait imagery, he again presses the button and opens his eyes.

During BOLD acquisition scans, participants imagined the same walking tasks (forward walking, backward walking, turning to the left, and turning to the right) as practiced overground. For each task, participants imagined walking two distances (4 and 8 meters for forward and backward gait; 2 and 3 revolutions for turns). Gait imagery tasks were completed with eyes closed and in a pseudo-random order. Each imagined gait bout was separated by an 11-second rest period in which eyes were open. It was necessary to have individuals open their eyes during rest to permit them to detect the visual cue of the upcoming task. In addition, monitoring when eyes were open and closed (via an MR compatible eye-tracker) provided another measure of adherence to the task. In a small number of runs, participants opened their eyes at inappropriate times. When this occurred, we stopped the scan, repeated the instructions, and started the scan anew. Inclusion of the eyes open rest tasks may have altered the baseline BOLD signal during fMRI runs. However, the same rest tasks were included for all runs (i.e. imagined standing and imagined gait) and for both groups. During gait imagery, participants tapped their index finger on a custom made MRI compatible button box (Mag Design and Engineering, Redwood City, CA, USA) once at the beginning and once at the end of each gait task to log the start and finish of each imagery epoch (Figure 1). Timing of each button press was recorded and used for post hoc assessment of imagery times and event related design modeling. By measuring the time taken to imagine walking short and long distances, we could assess the degree to which participants adhered to the tasks during scans. Participants were instructed to imagine in a first person perspective, and not to count steps.

In a third, four minute long T2*-weighted scan, participants alternated between imagined upright standing (20 seconds) and rest (11 seconds). For this scan a tactile cue on the leg indicated the end of imagery. Tactile cues were used during imagined standing for logistical reasons. During imagined gait, participants' imagery times were self-selected (i.e. they stopped imagining and opened eyes after completing the gait task). During imagined standing, however, it was necessary to notify the participant when to stop imagining. The tactile cue was modeled into the GLM to account for any associated changes in BOLD signal. Participants' eyes were closed during imagery of standing and open during rest, analogous to the imagined gait task. Thus, all imagery (imagined gait and imagined standing) was conducted with eyes closed.

Stimuli were projected onto a screen behind the participant and were viewed via a mirror mounted on the head coil. Instructions were presented using E-Prime v1.0 (Psychology Software Tools, Inc, Sharpsburg, PA). An MRI-compatible eye tracker documented that the eyes were closed and open at appropriate times. Presence of tremor of the eyes, head, lower legs and hands during scans was assessed qualitatively by observation. Two participants were excluded due to tremor during the scans (See Results).

### 4 FMR pre-processing

Functional data were preprocessed using Brain Voyager (v. 2.4.0.2000, 32-bit). The first two volumes from each imaging run were discarded for all trials. 3D motion correction was completed via sinc-interpolation. Slice scan time differences were corrected via sinc interpolation, and data were high pass filtered (the lowest two cycles were removed). Functional scans were then coregistered (i.e. spatially aligned) to participant-specific T1-weighted images which were normalized to Talairach space [41]. Task conditions were modeled with an event-related design with event length equal to the time taken to imagine that particular task and convolved with the canonical hemodynamic response function, which accounts for the delayed cerebral blood oxygenation changes following changes in neuronal activity. In addition to the 3D motion correction, any scan in which more than 2 mm or 2° of motion in any direction was detected was not included in the analysis. Neither maximum head movement (p = 0.56), nor standard deviation of head movement (p = 0.91) during scans differed between groups.

### 5 Region of interest (ROI) analysis

**Figure 2:**
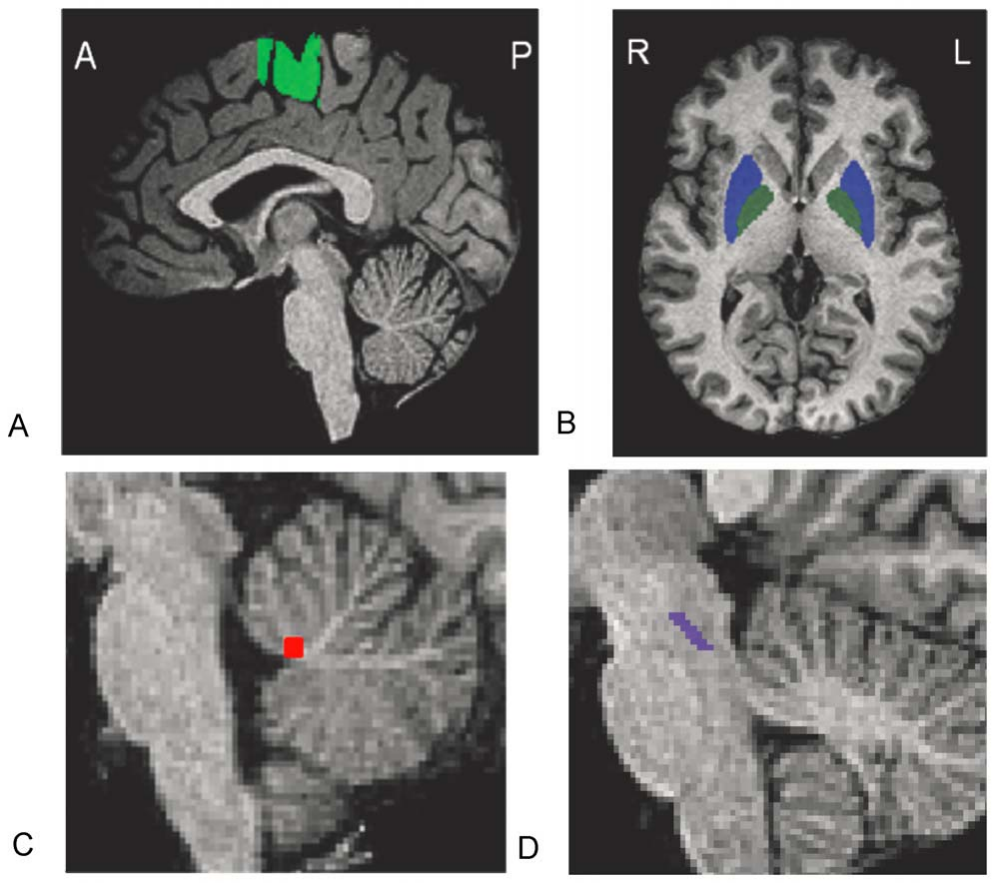
Regions of interest. Regions were identified for each individual separately based on standard definitions (see Methods). Shown are examples of regions defined for four subjects: supplementary motor area (a), putamen and globus pallidus (b), cerebellar locomotor region (c), and mesencephalic locomotor region (d). A-Anterior; P-Posterior; R-Right; L-Left.

BOLD signal was analyzed only within a-priori ROIs. We chose this approach for three reasons. First, ROIs can be identified manually on each participant more precisely than using a standardized template. Participant-specific region identification is particularly important for investigations into activity of small target regions. Second, a-priori identification of ROIs limits the need for multiple comparisons with respect to whole-brain analyses. Finally, the a priori selection of ROIs allow for a hypothesis driven approach to understanding locomotor dysfunction in PD. We chose nine ROIs (bilateral SMA, bilateral putamen, bilateral GP, bilateral MLR, and CLR) due to their link to both human locomotion [20], [42]–[44] and dysfunction in individuals with PD [14], [18], [21], [45]–[51]; particularly in people who experience freezing [14], [50], [52], [53]. Since our tasks of interest involved imagined movements, primary motor cortex was not included as a ROI, as this area does not typically responded to imagined motor tasks [16], [17], [54]. ROIs were identified manually for each participant on a high resolution MP-RAGE image warped to Talairach space [41]. A single operator, blinded to BOLD activation and group status, identified all ROIs. The SMA was identified as the midline grey matter superior to the cingulate sulcus. Parallel vertical lines through the anterior commissure (AC) and posterior commissure (PC) marked rostral and caudal boundaries [55]. The MLR was identified as a 54-voxel region of the brainstem lateral to the cerebellar peduncle decussation and medial lemniscus, including approximately the cuneate, subcuneate and pedunculopontine (PPN) nuclei [56], [57]. Rostral and caudal borders were based on previously defined borders of the PPN [57]–[60], The rostral border was defined at the intercollicular level [57], with the caudal border lying 6 mm inferior from this point, similar to Zrinzo and colleagues [59]. Because brainstem structures are difficult to identify on T1 scans, it is possible that the region identified excluded parts of the MLR or PPN, or included portions of other regions. However region identification was consistent across all subjects (both in location and size). The CLR was identified as a 72-voxel region of the midline white matter of the cerebellum, approximately rostral to the fastigial nuclei [43]. This region was chosen because it was shown specifically to be active during locomotion in previous gait imagery experiments in humans [30], and may be dysfunctional in those with PD [18]. Globus pallidus and putamen were identified using standard human atlases [61], [62]. Other non-motor regions (e.g. frontal and parietal areas) have been suggested to be related to freezing of gait (for review, see [63]). We did not include additional non-motor regions in order to limit the total number of ROIs and the need for multiple comparison correction. Average Talairach coordinates of each region are provided in Table 2, and examples of each ROI are shown in Figure 2. For comparison to other investigations, a non-linear transformation (mni2tal) was used to convert Montreal Neurologic Institute coordinates to Talairach coordinates (http://imaging.mrc-cbu.cam.ac.uk/imaging/MniTalairach).

**Table 2:** Mean (SD) of Talairach coordinates for each region of interest.

	Y		X		Z	
**Right SMA**	6.2	(0.8)	−11.6	(0.7)	53.3	(1.8)
**Left SMA**	−6.8	(1.1)	−11.4	(0.9)	52.9	(2.5)
						
**Right Putamen**	25.3	(1.2)	0.4	(1.6)	4.2	(1.1)
**Left Putamen**	−24.9	(1.0)	−0.4	(1.5)	3.9	(1.3)
						
**Right GP**	20.7	(1.3)	−3.8	(1.3)	2.7	(0.9)
**Left GP**	−20.2	(1.1)	−4.5	(1.2)	2.2	(1.1)
						
**Right MLR**	6.0	(0.9)	−26.0	(1.0)	−11.2	(1.4)
**Left MLR**	−6.0	(0.9)	−26.0	(1.1)	−11.2	(1.4)
						
**CLR**	−0.3	(1.3)	−46.2	(2.5)	−25.2	(2.4)

### 6 Statistics

Analyses of variance (ANOVAs) assessed actual and imagined gait times in both groups. Pearson correlation statistics assessed the relationship between actual and imagined gait times.

A general linear model (GLM) was constructed for imagined gait BOLD data to determine how well the design matrix model explains data. Beta weight changes associated with 5 tasks (rest, forward, backward, turning left, and turning right) and incorporating 6-dimensional head motion were determined using the GLM. Beta weights represent how much of the BOLD signal change is attributed to each of the five tasks. The inclusion of 6-dimensional head motion in the GLM helps to account for alterations in signal due to movements of the brain. Beta weights were also calculated for imagined stand and rest. Due to potential changes in baseline BOLD signals across scans, imagined stand and imagined gait beta weights were normalized to rest BOLD signals from their respective fMRI scans. This was completed by subtracting rest beta weights collected during the imagined gait scan from imagined gait beta weights. Similarly, rest beta weights collected during the imagined stand scan were subtracted from the imagined stand beta weights. Differences between imagined tasks (gait and stand) and rest in each group was carried out via paired sample, two-sided t-tests, and was conducted for each ROI.

Analyses of covariance (ANCOVAs) were used to assess changes in beta weights during imagined gait across groups and across tasks for each ROI. To account for differences in motor severity across freezing and non-freezing groups, MDS-UPDRS-III was included as a covariate in the ANCOVA. Average gait imagery speed for each participant was also included as a covariate in the analysis. Spearman's ρ statistics were used to correlate beta weights in each ROI to behavioral measures (actual overground gait velocity and freezing severity [NFOG total score]). Statistical threshold for all analyses was set at p = 0.05.

## Results

### 1 Participants

fMRI data were collected from 26 participants with PD. Data from six participants were excluded due to head movement over 2 mm or 2°. Of these six, two also had severe hand tremor. Another individual was excluded because he later reported prior head trauma, and one individual was excluded due to poor imagery performance during the scan. Thus, 18 individuals with PD (nine FoG+ and nine FoG−) were included for further analysis. FoG+ and FoG− were of similar age. FoG+ had similar disease severity to FoG− based on MDS-UPDRS part III and Hoehn and Yahr stage. Imagery ability (KVIQ and GIQ) was similar across groups (Table 1).

### 2 Behavioral

**Figure 3:**
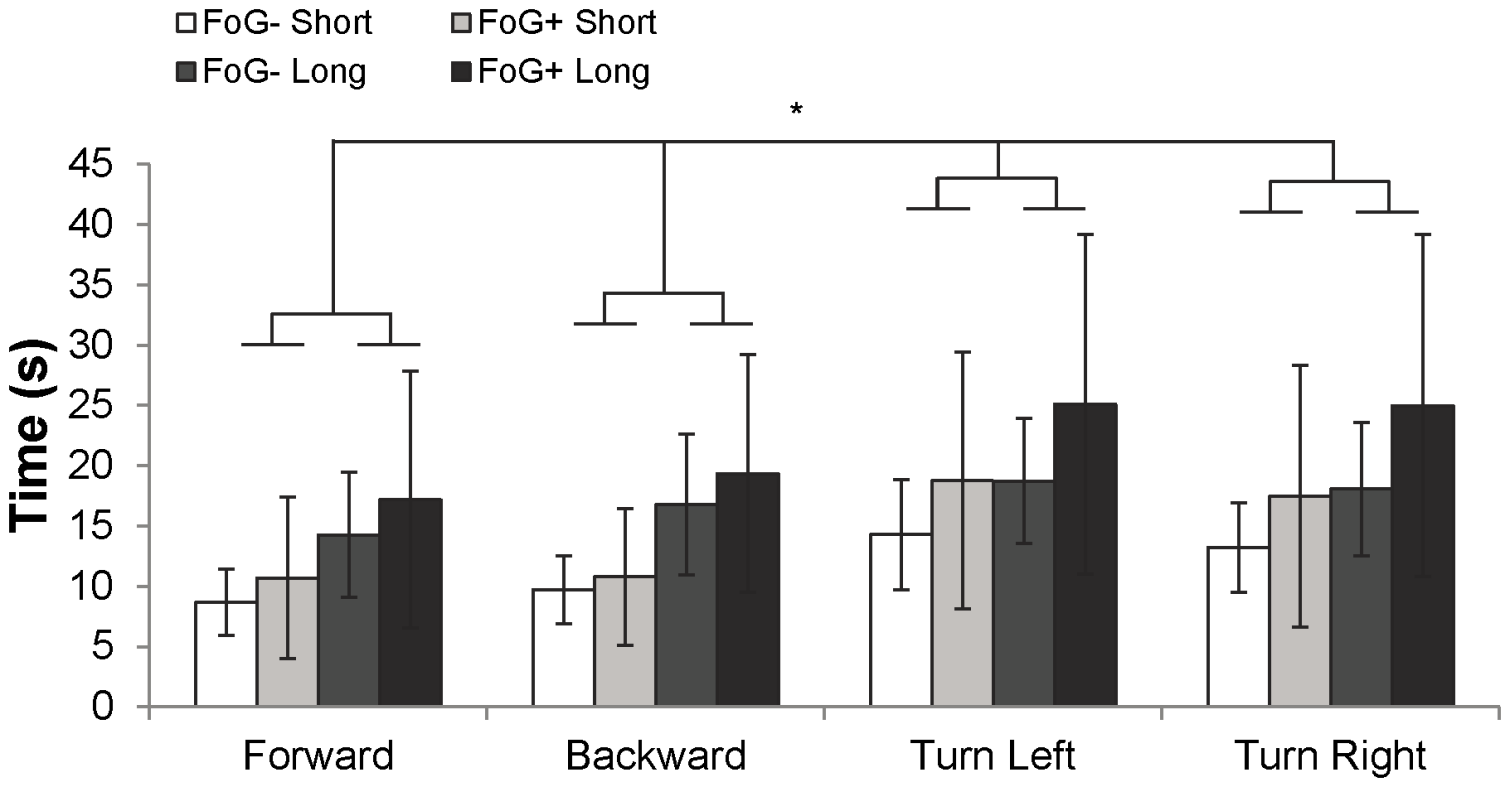
Gait imagery times in FoG− and FoG+ during short and long gait imagery tasks. “Long” gait imagery tasks took significantly longer than “short” gait imagery tasks (denoted by *, F_1,16_ = 34.6; p<0.001, repeated measures ANOVA). Differences between FoG+ and FoG− did not reach significance.

**Figure 4:**
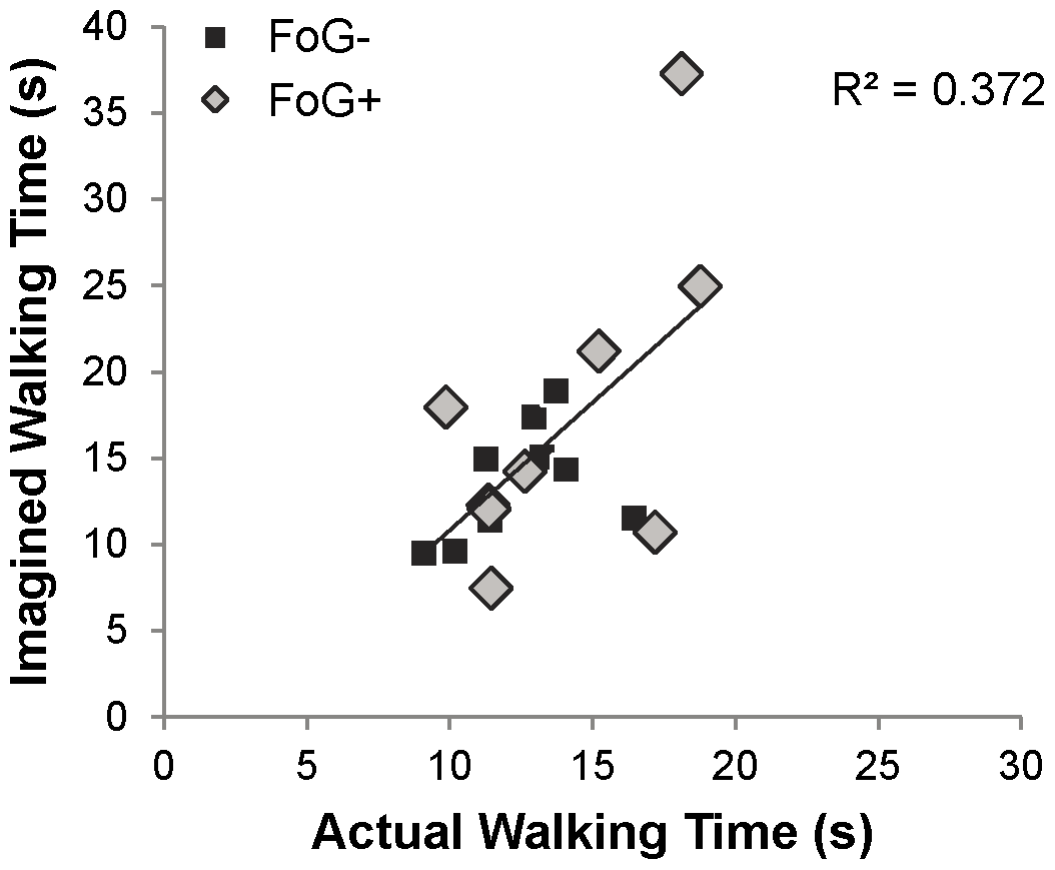
Correlation between actual and imagined walking times for freezers and non-freezers. Correlation statistics represent all participants.

Actual overground walking times and gait imagery times were similar across groups (F_1,16_ = 1.26; p = 0.28 and F_1,16_ = 1.4; p = 0.25, respectively). No freezing events were reported during gait imagery via self-report. As expected, “long” gait imagery tasks took longer than “short” (F_1,16_ = 34.6; p<0.001, Figure 3). Gait imagery times were not quite significantly longer than actual overground gait times, (p = 0.053, paired sample t-test). Actual and imagined gait times correlated with each for all subjects (r = 0.61, p = 0.007, Figure 4). One participant, a freezer, exhibited considerably longer imagery time (37 seconds on average) than actual time (18 seconds on average). Though no freezing was noted during imagery, this participant may have experienced altered imagined gait with respect to overground walking. Therefore, we completed BOLD signal analyses with and without this participant; no changes were noted. Furthermore, inclusion of imagined walking speed in the ANCOVA attenuated the effect of this outlier. Therefore, all data presented herein include this individual.

### 4 Functional MRI

#### Imagined Stand

During imagined standing, beta weights were significantly less than zero in the MLR (FoG+ and FoG−), CLR (FoG−), and GP (FoG−), indicating a reduction in beta weights during imagined stand with respect to rest. In the FoG+ group, beta weights from the CLR were lower during imagined standing than in the FoG− group (Table 3). The Mini BESTest did not consistently correlate with beta weights during imagined stand. However, significant correlations were present between Mini BESTest and beta weights in the left and right putamen only in the FoG− group.

**Table 3:** Beta weights during imagined standing, normalized to rest.

	FoG-Mean (SD)#	FoG+Mean (SD)#	FoG−/FoG+ Comparison (p)$
Right SMA	−0.27(0.60)	−0.42(0.72)	0.56
Left SMA	−0.26(0.47)	−0.389(0.68)	0.70
Right Putamen	−0.48(0.67)	−0.23(0.64)	0.91
Left Putamen	−0.46(0.66)	−0.21(0.50)	0.63
Right GP	**−0.57(0.51)** *	−0.40(0.61)	0.65
Left GP	−0.22(0.41)	−0.12(0.27)	0.90
Right MLR	−0.20(0.47)	**−0.38(0.37)** *	0.24
Left MLR	**−0.48(0.32)** **	**−0.43(0.21)** **	0.71
CLR	**−0.40(0.22)** **	−0.01(0.33)	**0.01**

# Paired sample t-test comparing stand and rest beta weights.

$ Univariate ANCOVA with UPDRS as covariate.

*Significantly different from rest at the 0.05 level.

**Significantly different from rest at the 0.005 level.

Abbreviations: SMA: supplementary motor area, GP: globus pallidus, MLR: mesencephalic locomotor region, CLR: cerebellar locomotor region.

#### Imagined Gait

**Figure 5:**
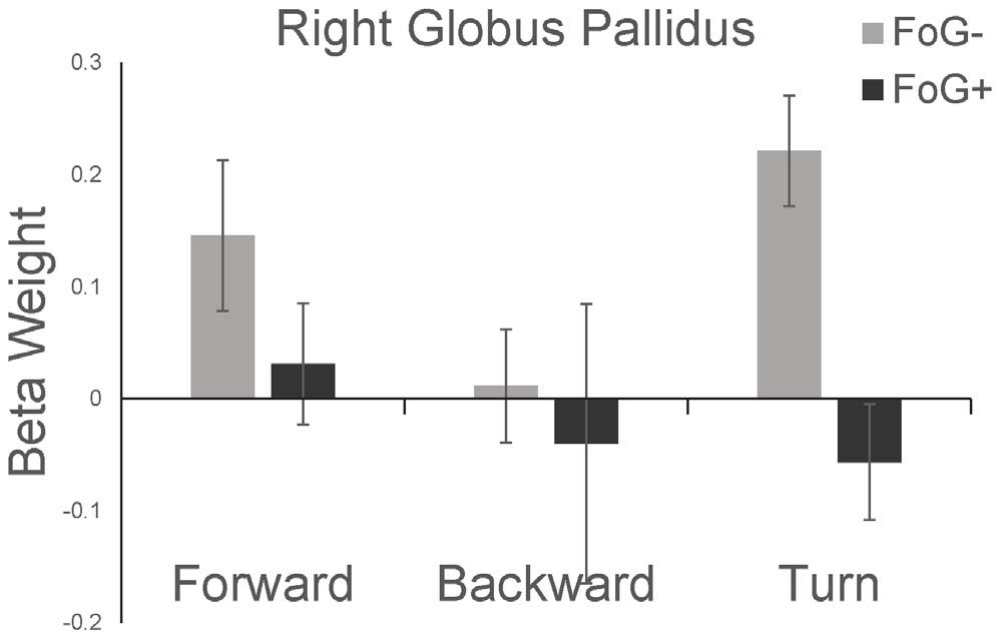
Mean Beta weights (with respect to rest) for FoG- and FoG+ during imagined walking in the right GP. A group effect, corrected for MDS-UPDRS and gait imagery speed, was noted such that FoG+ exhibited smaller BOLD signal than FoG- (p = 0.01). Error bars represent standard error of the mean.

**Figure 6:**
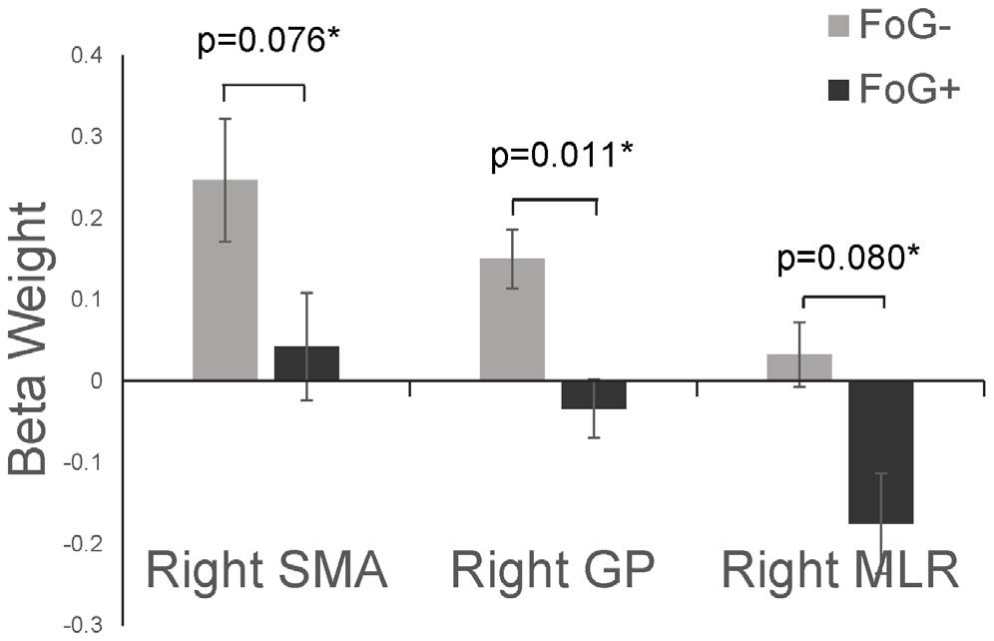
Mean beta weights (with respect to rest) for FoG− and FoG+ during imagined walking in the right SMA, right GP, and right MLR. Error bars represent standard error of the mean. *ANCOVA group differences after correcting for MDS-UPDRS and gait imagery speed.

Beta weights while imagining turning to the left and to the right did not differ in either group. Therefore, we combined data from imagined left and right turns for subsequent analyses. In the FoG− group, beta weights, normalized to rest, were significantly greater than zero in several locomotor regions, including the SMA, putamen, and GP, indicating increases in activity during imagined gait with respect to rest. In the FoG+ group, no regions were more active during imagined walking, and beta weights in the right MLR were lower during imagined walking than rest (Table 4). Despite similar ability to imagine walking (Table 1) and similar gait imagery times (Figure 3), a significant group effect was noted in the right GP, such that FoG+ exhibited smaller changes in signal than FoG− (Figure 5). Trends toward significant group effects were noted in the right SMA and right MLR (Table 4, Figure 6). No significant task or group by task interactions were observed. No consistent correlations were noted between beta weights during imagined walking and overground walking speeds. Of both groups, only the FoG+ group exhibited significant correlation of the right SMA beta weight with overground walking speed (Table 5).

**Table 4:** Mean (SD) beta weights and p-values for imagined walking in FoG− and FoG+, normalized to rest.

	FoG-mean (SD)#	FoG+mean (SD)#	FoG−/FoG+ comparison (p)$
Right SMA	**0.25 (0.23)** *	0.04 (0.12)	0.08
Left SMA	**0.23 (0.19)** **	0.10 (0.18)	0.11
Right putamen	**0.21 (0.25)** *	0.11 (0.35)	0.38
Left putamen	0.18 (0.31)	0.07 (0.25)	0.19
Right GP	**0.15 (0.11)** **	−0.03 (0.11)	**0.01**
Left GP	0.04 (0.11)	−0.05 (0.16)	0.80
Right MLR	0.03 (0.12)	**−0.18 (0.18)** *	0.08
Left MLR	0.01 (0.10)	−0.13 (0.23)	0.18
CLR	0.04 (0.18)	−0.04 (0.12)	0.20

# Paired sample t-test comparing gait and rest beta weights.

$ Repeated Measures ANCOVA with MDS-UPDRS and imagined gait velocity as co-variates: p-value for Group effects shown.

*Significantly different from rest at p = 0.05 level.

**Significantly different from rest at p = 0.01 level.

Abbreviations: SMA: supplementary motor area, GP: globus pallidus, MLR: mesencephalic locomotor region, CLR: cerebellar locomotor region.

**Table 5:** Correlation (Spearman's Rho and p-value) between imagined walking beta weights and actual overground walking speed.

	FoG−		FoG+	
	*ρ*	p	*ρ*	p
Right SMA	0.37	0.33	**−0.87**	**0.002**
Left SMA	0.42	0.27	−0.45	0.22
Right putamen	0.03	0.93	−0.47	0.21
Left putamen	0.05	0.90	0.05	0.9
Right GP	0.18	0.64	−0.32	0.41
Left GP	0.00	1.00	−0.42	0.27
Right MLR	0.17	0.67	0.17	0.67
Left MLR	−0.15	0.70	−0.02	0.98
CLR	0.53	0.14	0.00	1.00

Abbreviations: SMA: supplementary motor area, GP: globus pallidus, MLR: mesencephalic locomotor region, CLR: cerebellar locomotor region.

## Discussion

With respect to FoG−, FoG+ exhibited alterations in neural activity in the cerebellar locomotor region during imagined standing, and in the right GP during imagined walking. Other cortical and subcortical regions of the right side of the brain (MLR, SMA) also showed trends towards altered activity in FoG+ during imagined walking.

### 1 Imagined standing

During imagined standing, activity in most locomotor regions were similar to rest. When differences were observed (e.g. in the MLR), BOLD signal was lower than during rest. This result is in contrast to previous literature [24], [30], and is likely due to two differences in protocol. First, for both imagined standing and imagined gait bouts, participants had eyes closed during imagery, and open during rest periods, whereas previous investigations compared eyes closed imagined standing to eyes closed imagined lying. As noted above, we used this protocol to allow participants to read the text which informed their next task. In addition, by monitoring when participants' eyes were open and closed (via the eye tracker) we were able to gauge adherence to the task. Second, these previous studies were conducted on healthy young [30] and older [24] adults. Nonetheless, across group differences were noted in the CLR such that FoG− exhibited a larger change in BOLD signal than FoG+ between imagined standing and rest. Several recent reports have shown people with PD to have altered activity in the cerebellum with respect to healthy adults [64], for review, see [65]. Further, individuals who experience freezing have also been shown to exhibit neural changes in the cerebellum with respect to FoG. For example, two recent reports found reduced structural connectivity (measured via diffusion tensor imaging; DTI) between the cerebellum and the PPN (a sub-region of the MLR) in FoG+ compared to FoG− [50], [66]. These results, along with those of the current study suggest a possible relationship between cerebellar dysfunction and freezing.

### 2 Imagined walking

During imagined walking, FoG- exhibited increased neural activity with respect to rest in several neural regions including the SMA, putamen, and GP. This is consistent with previous literature which also demonstrates activity in locomotor regions during a number of gait like tasks in PD including imagined gait [14], [18], [67], actual gait [46], and stepping in a virtual reality environment [15]. Interestingly, FoG+ generally showed less neural activity during imagined gait than FoG−, despite having similar ability to imagine walking (Table 1) and similar gait imagery times (Figure 3). The significant reduction in neural activity in the GP in FoG+ with respect to FoG− is also partially consistent with previous reports. Shine and colleagues measured BOLD signal during alternating foot tapping in an immersive virtual reality environment. Freezers tapped their feet to move forward through the environment, and, in some cases, demonstrated lower limb motor blocks. In this study, and similar to results of the current report, BOLD signal in the GP (as well as the STN) was lower during lower limb motor blocks with respect to periods of non-motor blocks when controlling for changes in cognitive load during VR stepping.

We also observed trends toward reduced activity in other subcortical (MLR) and cortical (SMA) regions on the right side of the brain. Albeit preliminary, these results are in conjunction with a recent report investigating structural connectivity in FoG+ and FoG− using DTI. Fling and colleagues showed that FoG+ may have altered structural connectivity between the PPN and cortical/subcortical regions, particularly on the right side of the brain [66]. Other reports have also suggested that FoG may be related to changes in connectivity on the right side of the brain [63], [68]. For example, Tessitore and colleagues (2012) demonstrated FoG+ to have altered functional connectivity of the executive-attention network with respect to FoG−, particularly in the right hemisphere.

Only one previous report specifically investigated gait imagery in FoG+ and FoG− [14]. In this report, and similar to the current findings, FoG+ exhibited a trend toward reduced activity in the SMA. However, Snijders and colleagues also observed *increased* activity in the MLR in those who freeze. The discrepancy between results of Snijders and colleagues and the current study with respect to direction of MLR BOLD changes across groups may be due in part to different analysis techniques. We used a ROI-based analysis, while Snijders et al. used full-brain random effects general linear model. There are pros and cons to each method. An ROI analysis allows for more precise identification of regions to test specific hypotheses. However, for the ROI-base analysis used in the current study, all voxels were averaged within a region (we assume homogeneity within each ROI) and therefore subtle signal changes in subregions within the ROI could be missed. The differing results in the MLR may also have to do with the region definitions across studies. The region of interest identified as the MLR in the current study was manually identified for each participant based on stereotactic analyses of the PPN [57]–[59], and was, on average, slightly rostral to the area of increased activity described by Snijders and colleagues. Specifically, the approximate median position, in Talairach coordinates, of the area of differential activation in Snijders et al. was x = 0, y = −28, and z = −15 [14], whereas the midpoint of the MLR in the current study was x = +/−6, y = −26, z = −11. Though considerable effort has been put into identification of the MLR, and specifically the PPN, the precise location, identified via T1 weighted MRI, can be difficult to identify. Indeed, recent reports [69] have suggested the PPN may lie more caudal (approximate Talairach coordinates: x = +/−5, y = −29, z = −18) to the position noted in previous investigations [57]–[59]. Additional research into consistent identification of this region is warranted.

### 3 Complex Gait Imagery

Complex gait imagery tasks did not induce changes in beta weights with respect to simple gait imagery, despite the fact that actual gait is typically more dysfunctional in PD during complex tasks. Differences in actual and imagined locomotion may contribute to this finding. For example, imagined locomotion may require less balance and postural control than actual gait, limiting the differences in complexity across tasks. Despite these gait imagery limitations, Godde and colleagues showed a small increase in activity (14 voxels) in the left putamen during backward walking compared to forward walking, whereas we did not. Three factors may contribute to this discrepancy between studies. First, the previous report included a larger number of participants (n = 51), increasing their power to detect subtle across-task differences. Second, they had participants practice and imagine tandem backward walking while on a treadmill, while we had participants walk normally overground. Tandem walking is more difficult than normal gait, particularly in older adults [70], and may have led to a more pronounced BOLD signal change compared to imagined forward walking. Perhaps most importantly, they investigated healthy older adults, whereas we focused on people with PD, making direct comparisons across studies difficult [19].

### 4 Limitations

Functional neuroimaging during gait imagery permits investigation of brain pathophysiology that underlies gait tasks. However, this approach has several limitations. Although actual and imagined gait tasks activate similar brain circuits [29], inherent differences exist. Any task-related neuroimaging study depends upon accurate measurement and control of task performance. The covert nature of an imagined task makes this challenging. To ensure participants were able to effectively imagine movement, we screened for vividness of motor imagery (KVIQ score), and matched groups on ability to imagine both single limb movements (measured via the KVIQ), and imagined walking (measured via the GIQ). Furthermore, we obtained a measure of performance by comparing the length of time the participant imagined walking two different distances while in the scanner. Imagery times for longer distances were larger than short distances, suggesting participants were adhering to imagery tasks. This provided at least a rank order measure of performance of this covert task. Imagination of freezing during the imagery task also could confound task performance. However, no participants reported freezing events during gait imagery. Despite these various approaches to control imagery performance, we included imagined walking speed as a covariate in statistical analyses when comparing across groups as imagined walking at different speeds does alter BOLD responses [17], [47], [71]. FoG+ often have greater cognitive impairments than FoG− [72], [73]. To minimize this potential confound, we applied strict cognitive screening criteria for all participants (MMSE score had to exceed 26/30), and we matched FoG+ and FoG− on this measure. Differences in motor severity between FoG+ and FoG− also could be a confound. Therefore, we included MDS-UPDRS part III scores as a covariate in statistical analyses contrasting groups. Our relatively small sample size, limited in part to the strict cognitive and imagery screening, may have reduced our power to detect more modest changes in BOLD responses in some regions. Nevertheless, we had adequate power to detect changes in BOLD signal across groups in both the GP (during imagined walking), and in the CLR (during imagined standing). Finally, the lack of a healthy control group somewhat limits the interpretation of this study, as we cannot determine whether alterations in activity in FoG+ across tasks are similar to healthy adults.

## Conclusion

Individuals with PD who freeze exhibited altered activity in the cerebellum (during imagined stand), and in several regions of the right hemisphere (during imagined walking). These findings suggests those who freeze demonstrate distributed neural dysfunction, which may be more pronounced in the right hemisphere.
